# Design, synthesis, antimicrobial and cytotoxicity study on human colorectal carcinoma cell line of new 4,4′-(1,4-phenylene)bis(pyrimidin-2-amine) derivatives

**DOI:** 10.1186/s13065-018-0440-3

**Published:** 2018-06-25

**Authors:** Sanjiv Kumar, Siong Meng Lim, Kalavathy Ramasamy, Vasudevan Mani, Syed Adnan Ali Shah, Balasubramanian Narasimhan

**Affiliations:** 1 Faculty of Pharmaceutical Sciences, Maharshi Dayanand University, Rohtak, 124001 India; 2 Faculty of Pharmacy, Universiti Teknologi MARA (UiTM), 42300 Bandar Puncak Alam, Selangor Darul Ehsan Malaysia; 3 Collaborative Drug Discovery Research (CDDR) Group, Pharmaceutical Life Sciences Community of Research, Universiti Teknologi MARA (UiTM), 40450 Shah Alam, Selangor Darul Ehsan Malaysia; 4 Department of Pharmacology and Toxicology, College of Pharmacy, Qassim University, Buraidah, 51452 Saudi Arabia; 5 Atta-ur-Rahman Institute for Natural Products Discovery (AuRIns), Universiti Teknologi MARA, Puncak Alam Campus, 42300 Bandar Puncak Alam, Selangor Darul Ehsan Malaysia; 6 Faculty of Pharmacy, Universiti Teknologi MARA (UiTM), Puncak Alam Campus, 42300 Bandar Puncak Alam, Selangor Darul Ehsan Malaysia

**Keywords:** Pyrimidine molecules, Design, Synthesis, Antimicrobial, Cytotoxicity, HCT116

## Abstract

**Background:**

Pyrimidine molecules attracted organic chemists very much due to their biological and chemotherapeutic importance. Their related fused heterocycles are of interest as potential bioactive molecules so, we have designed and prepared a new class of 4,4′-(1,4-phenylene)bis(pyrimidin-2-amine) molecules and screened for their in vitro antibacterial, antifungal and cytotoxicity studies.

**Results:**

The structures of synthesized bis-pyrimidine molecules were confirmed by physicochemical and spectral means. The synthesized compounds were further evaluated for their in vitro biological potentials i.e. antimicrobial activity using tube dilution method and anticancer activity against human colorectal carcinoma (HCT116) cancer cell line by Sulforhodamine B assay.

**Conclusions:**

The biological study demonstrated that compounds **s7**, **s8**, **s11**, **s14**, **s16**, **s17** and **s18** have shown more promising antimicrobial activity with best MIC values than the cefadroxil (antibacterial) and fluconazole (antifungal) and compound **s3** found to have better anticancer activity against human colorectal carcinoma (HCT116) cancer cell line.
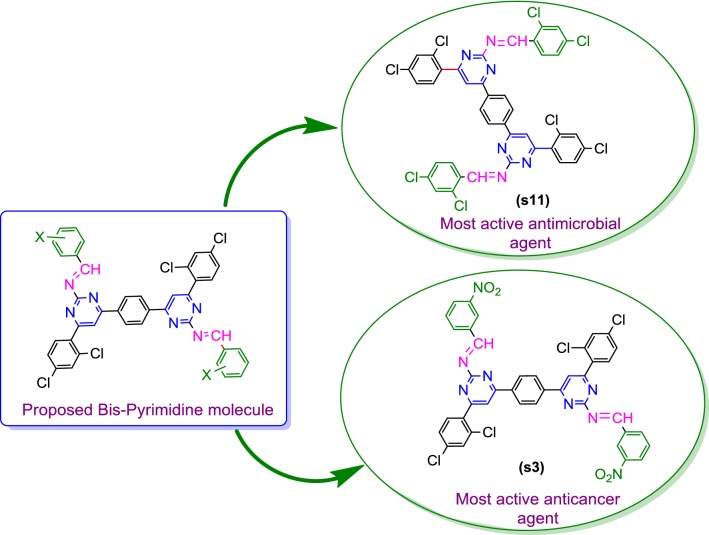

## Background

Among a wide variety of heterocyclic that have been explored for developing medicinally important molecules [1]. Pyrimidine derivatives attracted organic chemists very much due to their biological and chemotherapeutic importance especially the fused heterocycles are of interest as potential bioactive molecules. Pyrimidine derivatives are known to exhibit biological activities i.e. anticancer [2, 3], antiviral [4], anti-inflammatory [5], antimalarial [6], antibacterial [1, 7] and antifungal [8] etc. As pathogenic bacteria continuously evolve mechanisms of resistance to currently used antibacterial, so the discovery of novel and potent antibacterial drugs is the best way to overcome bacterial resistance and develop effective therapies [9].

**Fig. 1:**
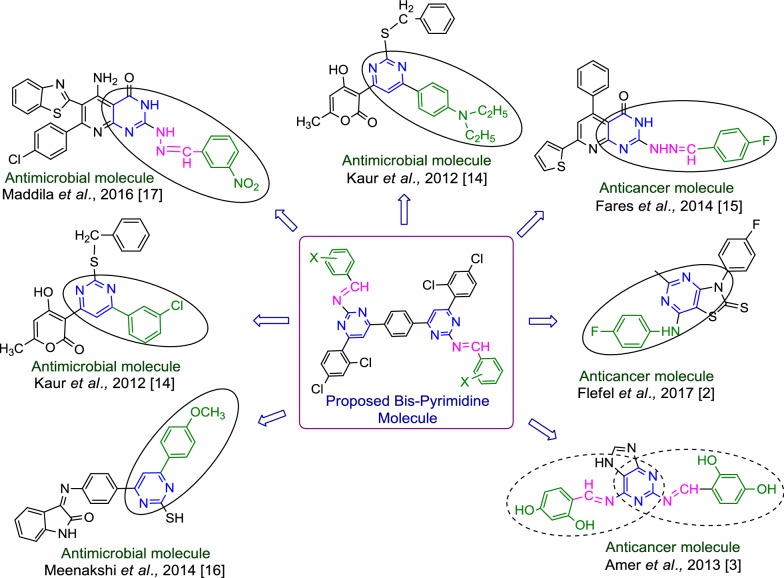
Design of pyrimidine molecules for antimicrobial and anticancer potential based on literature

**Fig. 2:**
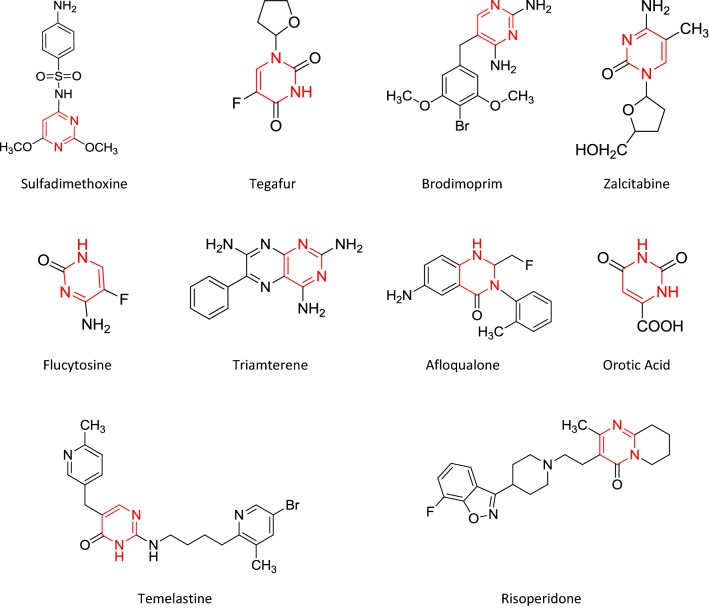
Selected marketed drug contains pyrimidine ring

Cancer is one of the most serious health problems all over the world and one of the leading causes of death. Thus, in the past for several decades, researchers have been struggling to find effective clinical approaches for the treatment of cancer and search for novel anticancer agents. Recently, accumulating evidences have illustrated that heterocyclic derivatives are considered to be the most promising molecules as leads for the discovery of novel synthetic drugs. In particular, substituted pyrimidines, present in the cores of many physiologically active molecules, display interesting therapeutic properties, especially antitumor activities with different bio targets and mechanisms by means of inhibiting several enzymes as well as modulating the activity of many receptors [10]. Pyrimidine is found as a core structure in a large variety of compounds that exhibit important biological activity, specifically pyrimidines known to inhibit *Pneumocystis carinii* (pc), *Toxoplasma gondii* (tg) of tumour cell lines in culture and the activity is attributed to inhibition of dihydrofolate reductase (DHFR) [11]. 2,4-Disubstituted and 2,4,6-trisubstituted pyrimidines have shown potent anticancer activity as CDK inhibitors, TNF-α inhibitors, Abl tyrosine protein kinase inhibitors, PI-3 kinase inhibitors, Akt kinase inhibitors and cytokines inhibitors [12]. Design of pyrimidine molecules for antimicrobial and anticancer potentials based on literature is presented in Fig. 1. Selected marketed drug contains pyrimidine ring presented in Fig. 2 [13].

On the basis of these observations, we here in report the synthesis, in vitro antimicrobial and cytotoxicity activities of 4,4′-(1,4-phenylene)bis(pyrimidin-2-amine) derivatives.

## Results and discussion

### Chemistry

**Scheme 1:**
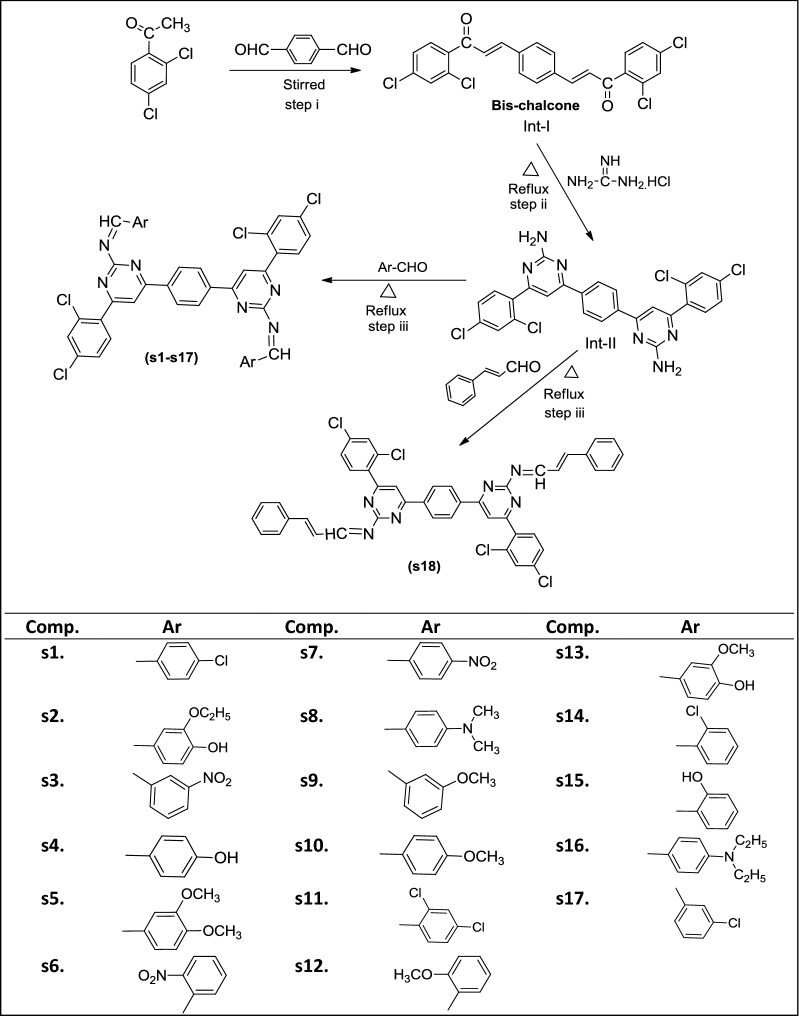
Synthesis of bis-pyrimidine molecules of 4,4′-(1,4-phenylene)bis(pyrimidin-2-amine)

Synthesis of the intermediate and target molecules was performed according to the reactions outlined in Scheme 1 (based on Claisen-Schmidt condensation). Initially, the bis-chalcone was prepared by the reaction of 1-(2,4-dichlorophenyl)ethanone and terephthalaldehyde. The cyclization of bis-chalcone (int-**I**) to yield bis-pyrimidine (int-**II**) was effected with guanidine hydrochloride. The reaction of bis-pyrimidine (int-**II**) with corresponding substituted aldehyde resulted in the formation of title compounds (**s1**–**s18**). The synthesized compounds were characterized by the determination of their physicochemical properties (Table 1) and spectral characteristics. The chemical structures of the synthesized 4,4′-(1,4-phenylene)bis(pyrimidin-2-amine)molecules **(s1**–**s18)** were established by ^1^H/^13^C-NMR, FT-IR, mass spectral studies and elemental analysis. The IR spectrum of bis-chalcone (**I)** showed the characteristic IR band at 1692.29 cm^−1^ which indicated the presence of –C=O group and characteristic bands at 3089.33 and 1596.22 cm^−1^ indicated the presence of C–H and C=C group in aromatic ring, respectively. The existence of Ar–Cl group in 3,3′-(1,4-phenylene)bis(1-(2,4-dichlorophenyl)prop-2-en-1-one) (**I)** was displayed by the existence Ar–Cl stretches in the scale of 752.59 cm^−1^ and characteristic bands at 2829.17 and 1461.71 cm^−1^ indicated the presence of C–H and C=C group in alkyl chain, respectively. 6,6′-(1,4-Phenylene)bis(4-(2,4-dichlorophenyl)pyrimidin-2-amine) (**II**) showed the characteristic bands at 3089.99 and 1598.31 cm^−1^ for the presence of C–H and C=C group in aromatic ring, respectively and characteristic bands at 3363.97 and 1692.49 cm^−1^ indicated the presence of –NH_2_ and N=CH str. The molecular structure of the intermediate-I and its cyclized products were further confirmed by proton–NMR spectral data. The ^1^H-NMR spectrum of intermediate-I showed two doublets at 7.59 ppm (*J* = 15.1 Hz) and 8.06 ppm (*J* = 15.1 Hz) indicating that the CH=CH group in the enone linkage is in a *trans*-conformation. The ^1^H-NMR spectrum of (**II)** showed a multiplet signals between 7.42 and 8.01 δ ppm confirming the cyclisation of the 3,3′-(1,4-phenylene)bis(1-(2,4-dichlorophenyl)prop-2-en-1-one) (**I)** to give 6,6′-(1,4-phenylene)bis(4-(2,4-dichlorophenyl)pyrimidin-2-amine) (**II**). The ^1^H-NMR spectrum of intermediate-**II** showed a sharp singlet at 7.09 δ ppm due to the NH_2_ protons and it also showed a sharp singlet at 7.85 δ ppm due to HC=C group, which confirmed the cyclization of the bis-chalcone into a bis-pyrimidine ring. The IR stretching vibrations at 733.88–750.53 cm^−1^ in the spectral data of synthesized derivatives **(s1**–**s18)** displayed the presence of halogen group (Ar–Cl) on the aromatic nucleus substituted at the *ortho*, *meta* and *para*-position. The existence of Ar–NO_2_ functional group in compounds **s3**, **s6** and **s7** was displayed by the existence of symmetric Ar–NO_2_ stretches in the scale of 1372.55–1373.82 cm^−1.^ The existence of an arylalkyl ether group (Ar–OCH_3_) in compounds, **s5**, **s9**, **s10**, **s12** and **s13** are established by the existence of an IR absorption band around 3088.33–3089.60 cm^−1^. The impression of IR stretching vibration at 3088.93–2972.97 cm^−1^ and 1599.67–1595.05 cm^−1^ in the spectral data of synthesized derivatives **(s1**–**s18)** specified the existence of C–H and C=C group, respectively. The appearance of IR stretching 1698.99–1663.17 cm^−1^ in the spectral data of synthesized derivatives **(s1**–**s18)** specified the existence of N=CH group. The impression of IR absorption band at 3461.41–3345.04 cm^−1^ in the spectral data **s2**, **s4**, **s13** and **s15** displayed the presence of Ar–OH group on the aromatic ring at *ortho* and *para* position. The multiplet signals between 6.77 and 8.34 δ ppm in proton-NMR spectra is indicative of aromatic proton of synthesized derivatives. The compounds, **s5**, **s9**, **s10**, **s12** and **s13** showed singlet at 3.84–3.85 δ ppm due to the existence of OCH_3_ of Ar–OCH_3_. The synthesized compounds showed singlet at 9.01–10.05 δ ppm due to the existence of N=CH in pyrimidine ring. Compounds showed singlet at 10.00–10.15 δ ppm due to the existence of –CH in pyrimidine ring. Compound **s8** showed singlet at 3.04 δ ppm due to existence of –N(CH_3_)_2_ at the *para* position. The compound **s16** showed quadrate at 3.38–3.49 δ ppm and triplet at 1.07–1.15 δ ppm due to presence of –N(C_2_H_5_)_2_ at *para* position. The ^13^C-NMR spectral data and elemental analysis studies of the synthesized pyrimidine derivatives were found within ± 0.4% of the theoretical results of synthesized compounds are given in the “Experimental section”.

**Table 1:**
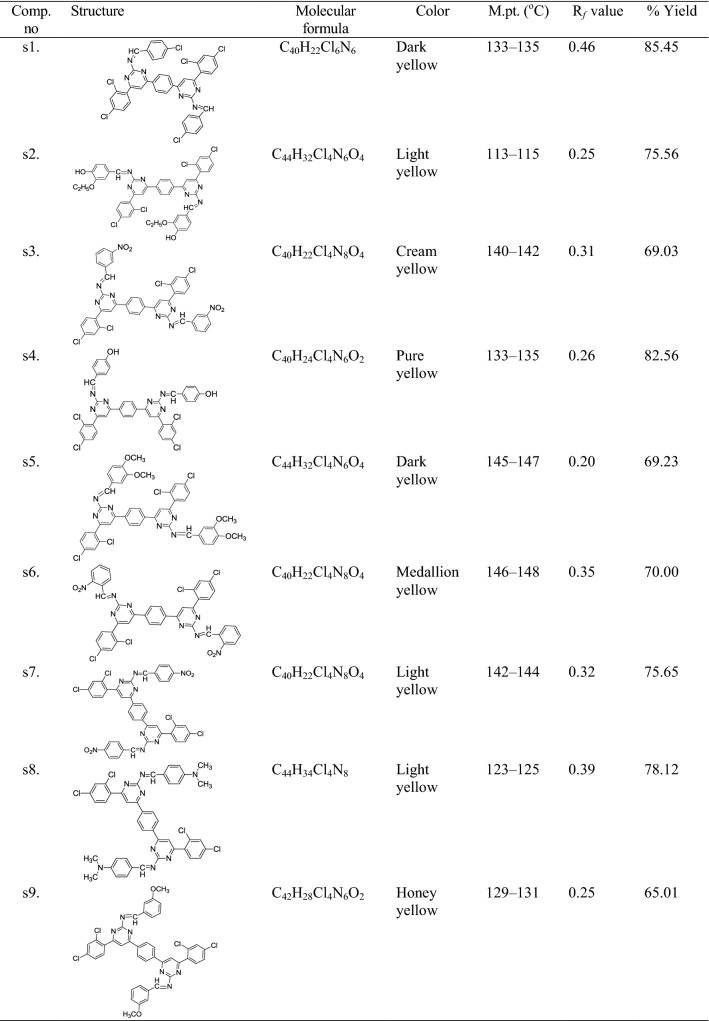

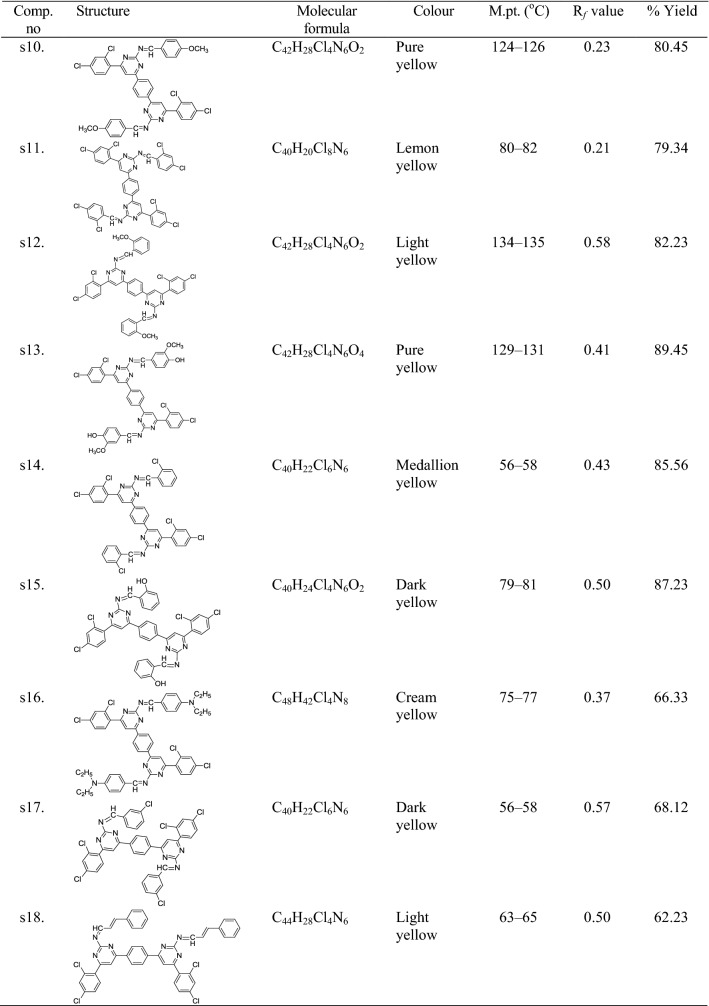
Physicochemical properties of the synthesized bis-pyrimidine molecules

### In vitro antimicrobial activity

Antimicrobial screening of synthesized 4,4′-(1,4-phenylene)bis(pyrimidin-2-amine) molecules against Gram positive and Gram negative bacterial and fungal strains was done by tube dilution technique. Antimicrobial activity results indicated (Table 2) particularly; compounds, **s7**, **s8**, **s11**, **s14**, **s16**, **s17** and **s18** have shown more promising antimicrobial activity as compared to standard drugs cefadroxil (antibacterial) and fluconazole (antifungal) while other derivatives are moderately active. In the case of Gram positive bacterial study, compound **s11** (MIC_*sa*_ = 0.14 µmol/mL, MIC_*bc*_ = 0.07 µmol/mL) was found to be most potent one against *S. aureus* and *B. cereus.* In the case of Gram negative bacterial study, compound **s7** and **s18** displayed appreciable antibacterial activity against *Providencia rettgeri* with MIC value of 0.08 µmol/mL. Compound **s8 (**MIC_*pa*_ = 0.15 µmol/mL) exhibited excellent activity against *Pseudomonas aeruginosa* and compound **s11** showed good antibacterial activity against *Salmonella typhi* and *Escherichia coli* with the MIC values of 0.29 and 0.23 µmol/mL, respectively. The antifungal screening results demonstrated that compounds **s11** displayed appreciable antifungal activity against *Aspergillus niger* and *Aspergillus flavus* with the MIC values of 0.58 and 0.14 µmol/mL, respectively. Compounds, **s14** and **s17** (MIC_*af*_ = 0.31 µmol/mL) were found to be most potent ones against *Aspergillus fumigatus* and compound **s16** (MIC_*af*_ = 0.14 µmol/mL) was found to be most effective one against *Aspergillus flavus.* The antimicrobial screening results of synthesized molecules (**s7**, **s8**, **s11**, **s14**, **s16**, **s17** and **s18**) have more than standard drugs and may be used as a lead compound to discover novel antimicrobial scaffolds.

**Table 2:** Antimicrobial activity results of synthesized bis-pyrimidine molecules

Compound no.	Antimicrobial activity (MIC = µmol/mL)						Fungal species		
	Bacterial species								
	Gram positive		Gram negative						
	*S.A.*	*B.C.*	*S.T.*	*P.A.*	*E.C.*	*P.R.*	*A.N.*	*A.F.* ^a^	*A.F.* ^b^
**s1.**	0.16	0.08	–	–	0.25	0.16	0.63	1.26	–
**s2.**	2.36	1.18	–	2.36	–	2.36	2.36	1.18	0.59
**s3.**	0.61	0.61	–	–	–	0.15	0.61	1.22	0.61
**s4.**	0.16	0.08	0.33	0.16	0.26	0.16	2.63	0.66	0.16
**s5.**	0.59	0.59	–	–	–	0.15	–	1.18	1.18
**s6.**	–	0.15	2.44	1.22	0.61	–	2.44	0.61	0.15
**s7.**	0.61	–	1.22	0.61	2.44	0.08	–	1.22	–
**s8.**	–	1.23	0.31	0.15	0.61	–	2.46	0.61	0.15
**s9.**	0.16	0.08	0.32	0.16	0.25	0.16	–	–	0.32
**s10.**	1.27	0.63	1.27	2.54	–	0.63	0.63	1.27	0.32
**s11.**	0.14	0.07	0.29	1.16	0.23	0.14	0.58	–	0.14
**s12.**	1.27	0.63	–	2.54	–	0.63	–	1.27	0.32
**s13.**	1.22	0.61	–	–	1.22	0.61	1.22	1.22	–
**s14.**	2.51	1.26	–	1.26	–	2.51	–	0.31	0.63
**s15.**	0.66	0.66	–	–	0.66	0.16	0.66	0.33	–
**s16.**	0.57	0.57	–	–	0.57	0.14	2.30	0.57	0.14
**s17.**	–	0.16	2.51	1.26	0.63	1.26	0.63	0.31	1.26
**s18.**	0.64	–	1.28	0.64	2.56	0.08	1.28	–	0.32
Acetone	NA	NA	NA	NA	NA	NA	NA	NA	NA
Broth control	NG	NG	NG	NG	NG	NG	NG	NG	NG
***Std.***	0.34^c^	0.34^c^	0.68^c^	0.68^c^	0.34^c^	0.68^c^	0.82^d^	0.82^d^	0.82^d^

*S.A.*: *Staphylococcus aureus*; *B.C.*: *Bacillus cereus*; *S.T.*: *Salmonella typhi*; *P.A.*: *Pseudomonas aeruginosa*; *E.C.*: *Escherichia coli*; *P.R.*: *Providencia rettgeri*; *A.N.*: *Aspergillus niger*

^a^*A.F.*: *Aspergillus fumigatus*; ^b^*A.F.*: *Aspergillus flavus*; Resistant (–); *NA* no activity, *NG* No growth

***Std.***: Cefadroxil^c^; Fluconazole^d^

### In vitro anticancer activity

**Fig. 3:**
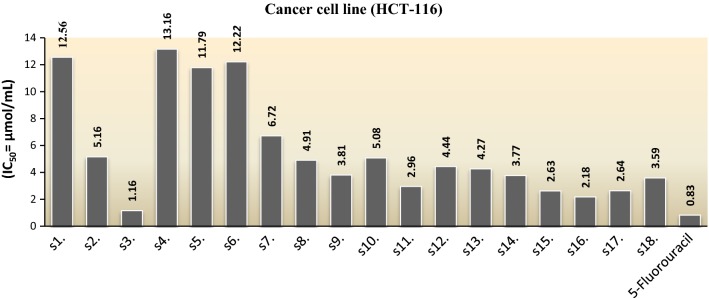
Anticancer screening results of synthesized molecules against cancer cell line

The in vitro anticancer activity of synthesized 4,4′-(1,4-phenylene)bis(pyrimidin-2-amine) molecules was carried out against human colorectal cancer cell line [HCT-116 (ATCC CCL-247)] and compared with 5-fluorouracil (reference drug) and the results of anticancer studies have been presented in Table 3, Fig. 3. Anticancer screening results revealed that in general pyrimidine exhibited good anticancer potential against human colorectal cancer cell line, especially, compound **s3** (IC_50_ = 1.16 µmol/mL) displayed anticancer activity comparable to the reference drug 5-fluorouracil (IC_50_ = 0.83 µmol/mL).

**Table 3:** Anticancer activity results of the synthesized bis-pyrimidine molecules

Anticancer activity (IC_50_ = µmol/mL)			
Compound no.	Cancer cell line (HCT-116)	Compound no.	Cancer cell line (HCT-116)
**s1.**	12.56	**s10.**	5.08
**s2.**	5.16	**s11.**	2.96
**s3.**	1.16	**s12.**	4.44
**s4.**	13.16	**s13.**	4.27
**s5.**	11.79	**s14.**	3.77
**s6.**	12.22	**s15.**	2.63
**s7.**	6.72	**s16.**	2.18
**s8.**	4.91	**s17.**	2.64
**s9.**	3.81	**s18.**	3.59
**5-Fluorouracil**	0.83	**5-Fluorouracil**	0.83

*HCT-116* human colorectal carcinoma

### Structure–activity relationship

**Fig. 4:**
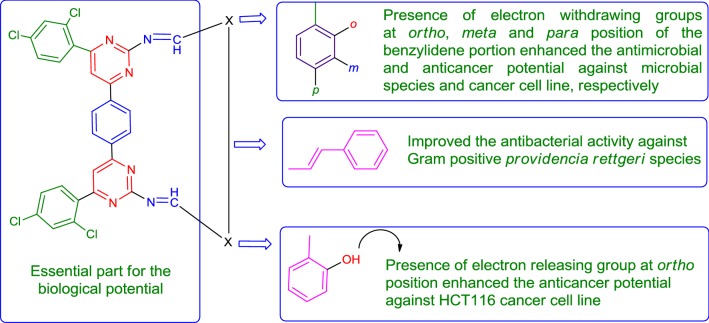
Structural requirements for the antimicrobial and anticancer activities of synthesized bis-pyrimidine molecules

From the in vitro antibacterial, antifungal and anticancer results, the structure–activity relationship of synthesized 4,4′-(1,4-phenylene)bis(pyrimidin-2-amine) molecules (SAR, are presented in Fig. 4).

From structure activity relationship study, we may conclude that different structural requirements are required for a compound to be effective against different targets. The aforementioned facts are supported by the earlier research findings [3, 14, 16, 17].

## Experimental section

Preliminary materials (glasswares, chemicals etc.) for the research work were obtained from commercial sources [Loba Chemie, Pvt Ltd. Mumbai, India; Central Drug House (CDH) Pvt. Ltd., New Delhi, India and HiMedia Laboratory Pvt. Ltd., Delhi, India] used without further purification. All reactions were monitored by thin-layer chromatography on 0.25 mm silica gel (Merck) plates, using benzene, chloroform: methanol as mobile phase and spots were observed by exposure to iodine vapours. Melting points of synthesized 4,4′-(1,4-phenylene) bis(pyrimidin-2-amine) molecules was determined in open capillary tube technique. Mass spectra of the synthesized compounds were recorded on Waters Micromass Q-ToF Micro instrument. An infrared spectrum (IR) was recorded (KBr-pellets) in Bruker 12060280, Software: OPUS 7.2.139.1294 spectrometer. ^1^H-NMR and ^13^C-NMR were recorded at 600 and 150 MHz respectively on Bruker Avance III 600 NMR spectrometer by appropriate deuterated solvents. The results are conveyed in parts per million (*δ*, ppm) downfield from tetramethyl silane (internal standard). ^**1**^H-NMR spectral details of the synthesized derivatives are represented with multiplicity like singlet (s); doublet (d); triplet (t); quartet (q); multiplet (m) and the number hydrogen ion. Elemental analysis of the synthesized 4,4′-(1,4-phenylene) bis(pyrimidin-2-amine) molecules was obtained by Perkin–Elmer 2400 C, H and N analyzer. All the compounds gave C, H and N analysis within ± 0.4% of the theoretical results.

## General procedure for the synthesis of 4,4′-(1,4-phenylene)bis(pyrimidin-2-amine) derivatives (s1–s18)

### Step i: Synthesis of 3,3′-(1,4-phenylene)bis(1-(2,4-dichlorophenyl)prop-2-en-1-one) (Int-I)

A mixture of 1-(2,4-dichlorophenyl)ethanone (0.02 mol) and terephthalaldehyde (0.01 mol) were stirred in ethanol (10–20 mL) for 2–3 h and 10 mL 40% sodium hydroxide solution was added drop wise with constant stirring at room temp till a light brown mass was obtained. Then the mixture was kept overnight at room temperature and the contents were poured on crushed ice and acidified with dilute hydrochloric acid, which resulted in the precipitation of chalcone. The crude 3,3′-(1,4-phenylene)bis(1-(2,4-dichlorophenyl)prop-2-en-1-one) was filtered, dried and recrystallized from methanol [18, 19].

### Step ii: Synthesis of 6,6′-(1,4-phenylene)bis(4-(2,4-dichlorophenyl)pyrimidin-2-amine) (Int-II)

To a mixture of 3,3′-(1,4-phenylene)bis(1-(2,4-dichlorophenyl)prop-2-en-1-one) (0.01 mol) (*synthesized in previous step*-*i*) and potassium hydroxide (0.01 mol) in 80 mL absolute ethanol, 40 mL 0.50 M solution of guanidine hydrochloride in ethanol was added. After addition, the mixture was refluxed for 4–5 h (50 °C). The progress of reaction was monitored by TLC and the reaction mixture was cooled at room temperature and quenched with 20 mL of 0.5 M solution of hydrochloric acid in water. The reaction mixture was shaken to ensure mixing and concentrated to obtain solid which was recrystallized from ethanol [18].

### Step iii: Synthesis of final compounds (s1–s18)

A mixture of 6,6′-(1,4-phenylene)bis(4-(2,4-dichlorophenyl)pyrimidin-2-amine) (0.01 mol) (*synthesized in previous step*-*ii*) and substituted aldehyde (0.02 mol) were refluxed in minimum amount of ethanol in presence of small amount of glacial acetic acid as catalyst for 2–3 h (40 °C). The progress of reaction was monitored by TLC plates. The mixture was cooled and poured in ice cold water. The precipitated solid (different yellow color) was filtered and recrystallized with methanol.

### In vitro antimicrobial assay

The in vitro antimicrobial study of the synthesized 6,6′-(1,4-phenylene)bis(4-(2,4-dichlorophenyl)pyrimidin-2-amine) molecules was evaluated against Gram positive bacteria [*Staphylococcus aureus* (ATCC 11632) and *Bacillus cereus* (MTCC 7350)], Gram negative bacteria [*Escherichia coli* ATCC 35218, *Pseudomonas aeruginosa* (ATCC 23564), *Salmonella typhi* (ATCC 15499) and *Providencia rettgeri* (MTCC-8099)] and fungal strains-*Aspergillus niger* (MTCC-281)*; Aspergillus fumigatus* (2483)*; Aspergillus flavus* (1783) by tube dilution method [20]. The stock solution was prepared for the test compounds (**s1**–**s18**) and reference drugs (cefadroxil and fluconazole) in acetone to get a concentration of 100 µg/mL and this stock solution was used for further tube dilution with six concentration of 20, 10, 5.0, 2.5, 1.25, 0.625 µg/mL for the antimicrobial study [21]. Dilution of test and standard compounds were prepared double strength nutrient broth—I.P (antibacterial) and sabouraud dextrose broth—I.P (antifungal). The samples were incubated at 37 ± 1 °C for 24 h (bacteria), 25 ± 1 °C for 7 days (*A. niger*), 30 ± 1 °C for 15 days (*A. flavus*), 35 ± 1 °C for 72 h (*A. fumigates*) respectively and results were recorded in terms of MIC (The lowest concentration of test substances which inhibited microbial growth) are presented in the Table 2.

### In vitro anticancer assay

The in vitro cytotoxicity screening of synthesized 6,6′-(1,4-phenylene)bis(4-(2,4-dichlorophenyl)pyrimidin-2-amine) molecules was determined against human colorectal carcinoma [HCT-116 (ATCC (American Type Culture Collection) CCL-247)] cancer cell line using Sulforhodamine-B (SRB) assay. In this study, the cells were fixed with trichloroacetic acid and then stained with 0.4% (w/v) Sulforhodamine B mixed with 1% acetic acid. Unbound dye was discarded by five washes of 1% acetic acid solution and protein-bound dye was solublised with 10 mM Tris base for confirmation of optical density in a computer-interfaced, 96-well microtiter plate reader. The anticancer activity results recorded in IC_50_ value [22].

## Spectral characteristics of the synthesized pyrimidine compounds (s1–s18)

### 6,6′-(1,4-Phenylene)bis(*N*-(4-chlorobenzylidene)-4-(2,4-dichlorophenyl)pyrimidin-2-amine) (**s1**)

IR (KBr, cm^−1^): 3088.93 (C–H str.), 1596.06 (C=C str.), 1665.82 (N=CH str.), 1332.11 (C–N str.), 735.72 (Ar–Cl); MS ES + (ToF): *m/z* 797 [M^+^+1]; ^1^H-NMR (δ, DMSO-*d*_6_): 7.36–7.86 (m, 18H, Ar–H), 10.05 (s, 2H, N=CH), 10.22 (s, 2H, (CH)_2_ of pyrimidine ring); ^13^C-NMR (δ, DMSO-*d*_6_): 160.2, 145.7, 138.9, 136.2, 131.2, 130.2, 129.98, 129.94, 128.17, 128.13, 127.6; CHN: Calc. C_40_H_22_Cl_6_N_6_: C, 60.10; H, 2.77; N, 10.51; Found: C, 60.18; H, 2.71; N, 10.56.

### 4,4′-(((6,6′-(1,4-Phenylene)bis(4-(2,4-dichlorophenyl)pyrimidine-6,2-diyl))bis(azanylylidene)) bis(methanylylidene))bis(2-ethoxyphenol) (**s2**)

IR (KBr, cm^−1^): 2978.51 (C–H str.), 1595.22 (C=C str.), 1665.80 (N=CH str.), 1331.51 (C–N str.), 73616 (Ar–Cl), 1104.41 (C–O–C_2_H_5_, aralkyl ether), 3345.04 (O–H str.); MS ES + (ToF): *m/z* 849 [M^+^+1]; ^1^H-NMR (δ, DMSO-*d*_6_): 6.78–8.27 (m, 16H, Ar–H), 9.70 (s, 2H, N=CH), 10.03 (s, 2H, (CH)_2_ of pyrimidine ring), 3.98 (t, 4H, (CH_2_)_2_), 1.32 (d, 6H, (CH_3_)_2_); ^13^C-NMR (δ, DMSO-*d*_6_): 165.67, 164.08, 153.58, 147.66, 136.66, 137.17, 132.63, 132.57, 130.48, 129.48, 129.91, 128.84, 126.44, 112.44, 107.36, 64.4, 14.8; CHN: Calc. C_44_H_32_Cl_4_N_6_O_4_: C, 62.13; H, 3.79; N, 9.88; Found: C, 62.10; H, 3.68; N, 9.81.

### 6,6′-(1,4-Phenylene)bis(4-(2,4-dichlorophenyl)-*N*-(3-nitrobenzylidene)pyrimidin-2-amine) (**s3**)

IR (KBr, cm^−1^): 3088.09 (C–H str.), 1595.51 (C=C str.), 1665.33 (N=CH str.), 1337.08 (C–N str.), 734.25 (Ar–Cl); 1373.82 (C–NO_2_, str., NO_2_); MS ES + (ToF): *m/z* 819 [M^+^+1]; ^1^H-NMR (δ, DMSO-*d*_6_): 7.42–8.34 (m, 18H, Ar–H), 10.04 (s, 2H, N=CH), 10.15 (s, 2H, (CH)_2_ of pyrimidine ring); ^13^C-NMR (δ, DMSO-*d*_6_): 165.56, 164.35, 145.90, 138.92, 137.86, 137.49, 136.22, 135.37, 131.90, 130.49, 129.49, 129.88, 128.21, 127.61, 124.45; CHN: Calc. C_40_H_22_Cl_4_N_8_O_4_: C, 58.56; H, 2.70; N, 13.66; Found: C, 58.50; H, 2.73; N, 13.70.

### 4,4′-(((6,6′-(1,4-Phenylene)bis(4-(2,4-dichlorophenyl)pyrimidine-6,2-diyl))bis(azanylylidene)) bis(methanylylidene))diphenol (**s4**)

IR (KBr, cm^−1^): 3028.00 (C–H str.), 1595.49 (C=C str.), 1664.04 (N=CH str.), 1332.80 (C–N str.), 735.82 (Ar–Cl), 3461.39 (O–H str.); MS ES + (ToF): *m/z* 761 [M^+^+1]; ^1^H-NMR (δ, DMSO-*d*_6_): 7.33–8.34 (m, 18H, Ar–H), 9.79 (s, 2H, N=CH), 10.10 (s, 2H, (CH)_2_ of pyrimidine ring), 6.92 (s, 2H, Ar–OH); ^13^C-NMR (δ, DMSO-*d*_6_): 165.56, 164.36, 136.45, 136.40, 134.93, 130.24, 130.18, 129.94, 129.05, 128.03, 127.62, 116.30, 107.26; CHN: Calc. C_40_H_24_Cl_4_N_6_O_2_: C, 63.01; H, 3.17; N, 11.02; Found: C, 63.05; H, 3.19; N, 11.08.

### 6,6′-(1,4-Phenylene)bis(4-(2,4-dichlorophenyl)-*N*-(3,4-dimethoxybenzylidene)pyrimidin-2-amine) (**s5**)

IR (KBr, cm^−1^): 3027.69 (C–H str.), 1596.45 (C=C str.), 1663.75 (N=CH str.), 1332.00 (C–N str.), 735.35 (Ar–Cl), 3088.33 (C–O–CH_3_, aralkyl ether); MS ES + (ToF): *m/z* 849 [M^+^+1]; ^1^H-NMR (δ, DMSO-*d*_6_): 7.01–8.34 (m, 16H, Ar–H), 9.85 (s, 2H, N=CH), 10.10 (s, 2H, (CH)_2_ of pyrimidine ring), 3.84 (s, 12H, (OCH_3_)_4_); ^13^C-NMR (δ, DMSO-*d*_6_): 165.65, 164.36, 137.87, 136.95, 134.93, 134.93, 131.83, 130.43, 129.95, 128.22, 128.17, 127.62, 111.82, 107.25, 56.38; CHN: Calc. C_44_H_32_Cl_4_N_6_O_4_: C, 62.13; H, 3.79; N, 9.88; Found: C, 62.17; H, 3.75; N, 9.92.

### 6,6′-(1,4-Phenylene)bis(4-(2,4-dichlorophenyl)-*N*-(2-nitrobenzylidene)pyrimidin-2-amine) (**s6**)

IR (KBr, cm^−1^): 3028.75 (C–H str.), 1595.16 (C=C str.), 1664.50 (N=CH str.), 1333.19 (C–N str.), 736.40 (Ar–Cl); 1372.97 (C–NO_2_ sym. str., NO_2_); MS ES + (ToF): *m/z* 819 [M^+^+1]; ^1^H-NMR (δ, DMSO-*d*_6_): 7.02–8.34 (m, 18H, Ar–H), 10.00 (s, 2H, N=CH), 10.10 (s, 2H, (CH)_2_ of pyrimidine ring); ^13^C-NMR (δ, DMSO-*d*_6_): 165.55, 164.36, 142.36, 137.87, 134.93, 132.66, 131.37, 130.27, 130.22, 129.99, 129.98, 128.16, 128.13, 127.61, 107.26; CHN: Calc. C_40_H_22_Cl_4_N_8_O_4_: C, 58.56; H, 2.70; N, 13.66; Found: C, 58.60; H, 2.74; N, 13.69.

### 6,6′-(1,4-Phenylene)bis(4-(2,4-dichlorophenyl)-*N*-(4-nitrobenzylidene)pyrimidin-2-amine) (**s7**)

IR (KBr, cm^−1^): 3088.00 (C–H str.), 1595.42 (C=C str.), 1664.00 (N=CH str.), 1334.07 (C–N str.), 735.84 (Ar–Cl), 1372.55 (C–NO_2_ sym. str., NO_2_); MS ES + (ToF): *m/z* 819 [M^+^+1]; ^1^H-NMR (δ, DMSO-*d*_6_): 7.02–8.34 (m, 18H, Ar–H), 10.00 (s, 2H, N=CH), 10.10 (s, 2H, (CH)_2_ of pyrimidine ring); ^13^C-NMR (δ, DMSO-*d*_6_): 165.55, 164.37, 163.17, 145.88, 142.74, 140.07, 137.86, 136.94, 134.93, 132.79, 131.84, 130.27, 129.94, 128.16, 128.10, 124.71, 107.1; CHN: Calc. C_40_H_22_Cl_4_N_8_O_4_: C, 58.56; H, 2.70; N, 13.66; Found: C, 58.61; H, 2.76; N, 13.70.

### 6,6′-(1,4-Phenylene)bis(4-(2,4-dichlorophenyl)-*N*-(4-(dimethylamino)benzylidene)pyrimidin-2-amine) (**s8**)

IR (KBr, cm^−1^): 3028.00 (C–H str.), 1590.10 (C=C str.), 1662.86 (N=CH str.), 1331.98 (C–N str.), 733.88 (Ar–Cl), 2826.15 (C–H str., –CH_3_); MS ES + (ToF): *m/z* 815 [M^+^+1]; ^1^H-NMR (δ, DMSO-*d*_6_): 6.77–8.34 (m, 18H, Ar–H), 9.67 (s, 2H, N=CH), 10.04 (s, 2H, (CH)_2_ of pyrimidine ring), 3.04 (s, 12H, (CH_3_)_4_); ^13^C-NMR (δ, DMSO-*d*_6_): 165.54, 164.36, 145.70, 142.74, 137.87, 136.40, 134.94, 131.37, 130.27, 130.18, 129.93, 128.16, 127.60, 111.54, 107.26, 40.15; CHN: Calc. C_44_H_34_Cl_4_N_8_: C, 64.72; H, 4.20; N, 13.72; Found: C, 64.76; H, 4.26; N, 13.74.

### 6,6′-(1,4-Phenylene)bis(4-(2,4-dichlorophenyl)-*N*-(3-methoxybenzylidene)pyrimidin-2-amine) (**s9**)

IR (KBr, cm^−1^): 3028.79 (C–H str.), 1595.05 (C=C str.), 1664.77 (N=CH str.), 1332.13 (C–N str.), 736.46 (Ar–Cl), 3089.60 (C–O–CH_3_, aralkyl ether); MS ES + (ToF): *m/z* 789 [M^+^+1]; ^1^H-NMR (δ, DMSO-*d*_6_): 7.03–8.34 (m, 18H, Ar–H), 10.04 (s, 2H, N=CH), 10.10 (s, 2H, (CH)_2_ of pyrimidine ring), 3.84 (s, 6H, (OCH_3_)_2_); ^13^C-NMR (δ, DMSO-*d*_6_): 165.55, 164.36, 145.86, 137.86, 136.94, 134.93, 139.79, 130.94, 129.99, 128.19, 128.12, 127.61, 116.6, 109.26, 56.8; CHN: Calc. C_42_H_28_Cl_4_N_6_O_2_: C, 63.81; H, 3.57; N, 10.63; Found: C, 63.85; H, 3.60; N, 10.68.

### 6,6′-(1,4-Phenylene)bis(4-(2,4-dichlorophenyl)-*N*-(4-methoxybenzylidene)pyrimidin-2-amine) (**s10**)

IR (KBr, cm^−1^): 3027.89 (C–H str.), 1595.38 (C=C str.), 1664.37 (N=CH str.), 1332.40 (C–N str.), 736.36 (Ar–Cl), 3088.96 (C–O–CH_3_, aralkyl ether); MS ES + (ToF): *m/z* 789 [M^+^+1]; ^1^H-NMR (δ, DMSO-*d*_6_): 7.02–8.34 (m, 18H, Ar–H), 10.05 (s, 2H, N=CH), 10.10 (s, 2H, (CH)_2_ of pyrimidine ring), 3.84 (s, 6H, (OCH_3_)_2_); ^13^C-NMR (δ, DMSO-*d*_6_): 165.57, 164.37, 163.50, 145.88, 139.94, 134.93, 132.73, 130.4, 129.88, 128.16, 128.13, 127.96, 114.9, 108.0, 52.2; CHN: Calc. C_42_H_28_Cl_4_N_6_O_2_: C, 63.81; H, 3.57; N, 10.63; Found: C, 63.85; H, 3.61; N, 10.70.

### 6,6′-(1,4-Phenylene)bis(*N*-(2,4-dichlorobenzylidene)-4-(2,4-dichlorophenyl)pyrimidin-2-amine) (**s11**)

IR (KBr, cm^−1^): 3025.92 (C–H str.), 1594.64 (C=C str.), 1663.17 (N=CH str.), 1330.99 (C–N str.), 734.93 (Ar–Cl); MS ES + (ToF): *m/z* 865 [M^+^+1]; ^1^H-NMR (δ, DMSO-*d*_6_): 7.28–8.02 (m, 16H, Ar–H), 10.02 (s, 2H, N=CH), 10.10 (s, 2H, (CH)_2_ of pyrimidine ring); ^13^C-NMR (δ, DMSO-*d*_6_): 165.59, 164.38, 163.52, 145.87, 137.80, 136.92, 137.78, 131.78, 130.94, 130.27, 129.80, 128.02, 128.05, 127.50, 100.00; CHN: Calc. C_40_H_20_Cl_8_N_6_: C, 55.33; H, 2.32; N, 9.68; Found: C, 55.338; H, 2.37; N, 9.72.

### 6,6′-(1,4-Phenylene)bis(4-(2,4-dichlorophenyl)-*N*-(2-methoxybenzylidene)pyrimidin-2-amine) (**s12**)

IR (KBr, cm^−1^): 3027.43 (C–H str.), 1592.13 (C=C str.), 1663.49 (N=CH str.), 1332.08 (C–N str.), 3088.50 (C–O–CH_3_, aralkyl ether), 735.94 (Ar–Cl); MS ES + (ToF): *m/z* 789 [M^+^+1]; ^1^H-NMR (δ, DMSO-*d*_6_): 6.96–7.87 (m, 18H, Ar–H), 9.20 (s, 2H, N=CH), 10.02 (s, 2H, (CH)_2_ of pyrimidine ring), 3.85 {s, 6H, (OCH_3_)_2_}; ^13^C-NMR (δ, DMSO-*d*_6_): 165.59, 164.37, 163.50, 145.88, 139.90, 135.98, 134.93, 132.73, 130.4, 129.80, 128.19, 128.13, 127.96, 115.9, 108.0, 55.2; CHN: Calc. C_42_H_28_Cl_4_N_6_O_2_: C, 63.81; H, 3.57; N, 10.63; Found: C, 63.85; H, 3.61; N, 10.68.

### 4,4′-(((6,6′-(1,4-Phenylene)bis(4-(2,4-dichlorophenyl)pyrimidine-6,2-diyl))bis(azanylylidene)) bis(methanylylidene))bis(2-methoxyphenol) (**s13**)

IR (KBr, cm^−1^): 3027.22 (C–H str.), 1596.30 (C=C str.), 1663.95 (N=CH str.), 1331.37 (C–N str.), 3461.41 (O–H str.), 3088.44 (C–O–CH_3_, aralkyl ether), 736.07 (Ar–Cl); MS ES + (ToF): *m/z* 821 [M^+^+1]; ^1^H-NMR (δ, DMSO-*d*_6_): 7.03–8.34 (m, 16H, Ar–H), 10.04 (s, 2H, N=CH), 10.10 (s, 2H, (CH)_2_ of pyrimidine ring), 3.85{s, 6H, (OCH_3_)_2_}; ^13^C-NMR (δ, DMSO-*d*_6_): 165.55, 164.36, 151.01, 149.01, 137.71, 136.94, 134.93, 132.78, 131.84, 130.94, 130.34, 129.88, 128.16, 128.05, 107.61, 61.35; CHN: Calc. C_42_H_28_Cl_4_N_6_O_4_: C, 61.33; H, 3.43; N, 10.22; Found: C, 61.38; H, 3.48; N, 10.27.

### 6,6′-(1,4-Phenylene)bis(*N*-(2-chlorobenzylidene)-4-(2,4-dichlorophenyl)pyrimidin-2-amine) (**s14**)

IR (KBr, cm^−1^): 2973.44 (C–H str.), 1599.67 (C=C str.), 1666.78 (N=CH str.), 1329.19 (C–N str.), 750.40 (Ar–Cl); MS ES + (ToF): *m/z* 797 [M^+^+1]; ^1^H-NMR (δ, DMSO-*d*_6_): 7.24–8.00 (m, 18H, Ar–H), 9.01 (s, 2H, N=CH), 10.10 (s, 2H, (CH)_2_ of pyrimidine ring); ^13^C-NMR (δ, DMSO-*d*_6_): 165.65, 164.35, 162.50, 146.75, 136.24, 134.37, 131.85, 130.31, 130.22, 130.18, 129.90, 129.20, 128.03, 120.02, 128.00, 127.88, 100.39; CHN: Calc. C_40_H_22_Cl_6_N_6_: C, 60.10; H, 2.77; N, 10.51; Found: C, 60.17; H, 2.80; N, 10.55.

### 2,2′-(((6,6′-(1,4-Phenylene)bis(4-(2,4-dichlorophenyl)pyrimidine-6,2-diyl))bis(azanylylidene)) bis(methanylylidene))diphenol (**s15**)

IR (KBr, cm^−1^): 2972.97 (C–H str.), 1598.70 (C=C str.), 1698.99 (N=CH str.), 1330.19 (C–N str.), 750.53 (Ar–Cl); 3360.91 (O–H str.); MS ES + (ToF): *m/z* 761 [M^+^+1]; ^1^H-NMR (δ, DMSO-*d*_6_): 7.27–7.99 (m, 18H, Ar–H), 9.99 (s, 2H, N=CH), 10.07 (s, 2H, (CH)_2_ of pyrimidine ring); ^13^C-NMR (δ, DMSO-*d*_6_): 165.55, 164.36, 137.78, 136.46, 130.31, 129.90, 128.93, 128.12, 117.08, 110.04; CHN: Calc. C_40_H_24_Cl_4_N_6_O_2_: C, 63.01; H, 3.17; N, 11.02; Found: C, 63.05; H, 3.19; N, 11.07.

### 6,6′-(1,4-Phenylene)bis(4-(2,4-dichlorophenyl)-*N*-(4-(diethylamino)benzylidene)pyrimidin-2-amine) (**s16**)

IR (KBr, cm^−1^): 2974.01 (C–H str.), 1590.98 (C=C str.), 1695.19 (N=CH str.), 1352.37 (C–N str.), 750.16 (Ar–Cl), 2826.51 (C–H str., –C_2_H_5_); MS ES + (ToF): *m/z* 871 [M^+^+1]; ^1^H-NMR (δ, DMSO-*d*_6_): 7.26–8.00 (m, 18H, Ar–H), 9.63 (s, 2H, N=CH), 10.00 (s, 2H, (CH)_2_ of pyrimidine ring), 3.38–3.49 {q, 8H, (CH_2_)_4_}, 1.07–1.15 {t, 12H, (CH_3_)_4_}; ^13^C-NMR (δ, DMSO-*d*_6_): 167.55, 164.36, 159.36, 136.42, 131.86, 130.30, 130.18, 129.91, 128.94, 127.49, 126.62, 124.49, 111.0, 44.43, 12.74; CHN: Calc. C_48_H_42_Cl_4_N_8_: C, 66.06; H, 4.85; N, 12.84; Found: C, 66.10; H, 4.90; N, 12.88.

### 6,6′-(1,4-Phenylene)bis(*N*-(3-chlorobenzylidene)-4-(2,4-dichlorophenyl)pyrimidin-2-amine) (**s17**)

IR (KBr, cm^−1^): 2974.44 (C–H str.), 1579.02 (C=C str.), 1693.29 (N=CH str.), 1328.36 (C–N str.), 750.11 (Ar–Cl); MS ES + (ToF): *m/z* 797 [M^+^+1]; ^1^H-NMR (δ, DMSO-*d*_6_): 7.25–8.03 (m, 18H, Ar–H), 10.00 (s, 2H, N=CH), 10.04 (s, 2H, (CH)_2_ of pyrimidine ring); ^13^C-NMR (δ, DMSO-*d*_6_): 165.55, 164.36, 136.91, 131.85, 131.77, 130.31, 130.23, 129.92, 129.21, 128.98, 128.10, 127.49, 126.65, 100.90; CHN: Calc. C_40_H_22_Cl_6_N_6_: C, 60.10; H, 2.77; N, 10.51; Found: C, 60.15; H, 2.80; N, 10.48.

### 4-(2,4-Dichlorophenyl)-6-(4-(6-(2,4-dichlorophenyl)-2-(((E)-3-phenylallylidene)amino) pyrimidin-4-yl)phenyl)-*N*-((E)-3-phenylallylidene)pyrimidin-2-amine (**s18**)

IR (KBr, cm^−1^): 2973.44 (C–H str.), 1597.13 (C=C str.), 1669.71 (N=CH str.), 1329.59 (C–N str.), 749.84 (Ar–Cl), 2829.12 (C–H str. aliphatic); ^1^H-NMR (δ, DMSO-*d*_6_): 7.45–8.04 (m, 20H, Ar–H), 7.55 {d, 2H, (CH)_2_ of N=CH}, 9.00 (s, 2H, (CH)_2_ of pyrimidine ring), 6.86 {t, 2H, (CH)_2_}, 7.34 {d, 2H, (CH)_2_}; ^13^C-NMR (δ, DMSO-*d*_6_): 167.89, 164.23, 163.9, 135.6, 134.69, 133.56, 130.20, 130.87, 129.88, 128.00, 128.34, 127.02, 127.90, 120.12, 110.1; CHN: Calc. C_44_H_28_Cl_4_N_6_: C, 67.53; H, 3.61; N, 10.74; Found: C, 67.51; H, 3.68; N, 10.77.

## Conclusion

In conclusion, we have described a simple and efficient protocol for the synthesis of new bis-pyrimidine molecules (**s1**–**s18**) with appreciable yields. The in vitro antibacterial, antifungal and anticancer potential of all the synthesized compounds were investigated. It is evident that synthesized compounds, **s7**, **s8**, **s11**, **s14**, **s16**, **s17** and **s18** have excellent antimicrobial activity and compound **s3** exhibited good anticancer activity. For the above compounds a significant improvement in their antibacterial and antifungal activities has been examined over the earlier reported compounds. The 4,4′-(1,4-phenylene)bis(pyrimidin-2-amine) molecules reported have a probability to emerge as a valuable lead series with great potential to be used as antibacterial, antifungal and anticancer agents and as promising candidates for further efficacy evaluation.
